# Estimating money laundering flows with a gravity model-based simulation

**DOI:** 10.1038/s41598-020-75653-x

**Published:** 2020-10-29

**Authors:** Joras Ferwerda, Alexander van Saase, Brigitte Unger, Michael Getzner

**Affiliations:** 1 Utrecht University School of Economics (U.S.E.), Kriekenpitplein 21-22, 3584 EC Utrecht, The Netherlands; 2 Vienna University of Technology – TU Wien, Karlplatz 13, 1040 Vienna, Austria

**Keywords:** Human behaviour, Statistics

## Abstract

It is important to understand the amounts and types of money laundering flows, since they have very different effects and, therefore, need different enforcement strategies. Countries that mainly deal with criminals laundering their proceeds locally, need other measures than countries that mainly deal with foreign illegal investments or dirty money just flowing through the country. This paper has two main contributions. First, we unveil the country preferences of money launderers empirically in a systematic way. Former money laundering estimates used assumptions on which country characteristics money launderers are looking for when deciding where to send their ill-gotten gains. Thanks to a unique dataset of transactions suspicious of money laundering, provided by the Dutch Institute infobox Criminal and Unexplained Wealth (iCOV), we can empirically test these assumptions with an econometric gravity model estimation. We use this information for our second contribution: iteratively simulating all money laundering flows around the world. This allows us, for the first time, to provide estimates that distinguish between three different policy challenges: the laundering of domestic crime proceeds, international investment of dirty money and money just flowing through a country.

## Introduction

Knowledge about the size and the effects of money laundering is important. Politicians and policymakers need this information to prioritize the problem and decide on the appropriate policy response. But also the type of money laundering matters. Money that is laundered from domestic crime needs domestically oriented anti-crime and anti-money laundering policy. Throughflows of crime money usually do not harm the domestic economy and need political will to cooperate with other countries as well as financial expertise to be discovered. Investment of dirty money in an economy, such as real estate being bought up by criminals, needs experts to check the justification of money spent on these objects^1^.

The economic and social effects of money laundering are manifold^2^ and became even more prominent since national governments are now obliged to estimate money laundering risks to fulfill international anti-money laundering regulations and standards^3^.

Money laundering cannot be measured directly by some easily accessible statistics since the whole purpose of money laundering is to disguise the criminal origin. Most prominent estimation models for money laundering are the so-called Walker- and Unger-models^4–8^. The Walker/Unger-model estimation procedures have received criticism from money laundering scholars on the lack of empirical foundation^9–11^, and from policy-makers on the lacking ability to distinguish between domestic and international money laundered in a country and money just flowing through a country (expressed in personal emails and conversations to the authors). The Walker/Unger-model estimation procedures only estimate the very first international transfer of money and ignore all international flows after that.

This paper has two main contributions to the literature. Former estimates used assumptions on which country characteristics money launderers are looking for when deciding the destination of their ill-gotten gains. For instance, it is assumed that bank secrecy and GDP attract money launderers, while corruption and conflict discourages money launderers to send their money to another country^7,8,12^. This paper tests these assumptions using a unique dataset of transactions suspicious of money laundering. To our knowledge, this is the first paper to unveil country preferences of money launderers. We use this information for our second contribution: simulating all money laundering flows around the world, providing the first estimates that distinguish between domestic money laundering, international investment of dirty money and money just flowing through.

Since the introduction of anti-money laundering policies, there has been a high demand for estimates of money laundering to justify the costs that are faced by public and private entities to chase the dirty money. Walker^6^, Schneider^13^, Unger^14^, and Baguella et al.^15^ all use an economic or econometric model to estimate the amount of money laundering. Walker^6^ was the first with a prototype model to estimate global money laundering. The Walker model is based on the gravity model from physics^8,16^. This type of gravity model estimates the worldwide allocation of dirty money that needs to be laundered. The portion of crime proceeds that are sent from country $i$ to $j$ depends on the ’attractiveness’ of country $j$ for laundering money, and the distance between country $i$ and $j$. Unger^14^ rejuvenated the Walker-model for the Netherlands by refining the distance indicator and updating the model. But, since the left side of the model (the amount of money laundering) is unknown, the coefficients of the attractiveness and distance factors were never estimated for the Walker or Unger models. The coefficients of the model are based on only an inspirational ad-hoc guess to calculate the amount of money laundering. The outcomes of these prototype models seem reliable when compared with other estimations^8^ but the actual model specification was never tested. Therefore the question remained open whether such a gravity-type model can properly estimate money laundering flows around the world.

The gravity model has been applied successfully for some decades to measure and predict all types of flows, like commuting, recreational traffic, patient flows to hospitals, migration, flows of buyers in shopping centers, and interregional and international trade. Perhaps it was therefore just a matter of time until the model was also applied to illegal flows, like heroin trade^17^, money flows to tax havens^18^, and money laundering^4,7,8^. The gravity model is inspired by Newton’s universal law of gravity, which states that the attraction between two objects depends on the mass of these objects and (the inverse of) their squared mutual distance and the gravity constant. Tinbergen^19^ laid the foundation for gravity models within the economic context. The gravity model as it is applied outside of physics stipulates that a flow from $i$ to $j$ is determined by stimulating or restraining forces relating to the specific flow between $i$ and $j$ and by supply conditions at the origin ($i$) and demand conditions at the destination ($j$). The gravity model in international trade generally looks like this^20^:

1 $${X}_{i,j}={\beta }_{0}{Y}_{i}^{{\beta }_{1}}{N}_{i}^{{\beta }_{2} }{Y}_{j}^{{\beta }_{3}}{N}_{j}^{{\beta }_{4} }{D}_{i,j}^{{\beta }_{5}}{P}_{i,j}^{{\beta }_{6}}$$

${X}_{i,j}$ represents the trade value between countries $i$ and $j$, $Yi$ and $Yj$ represent the Gross Domestic Product (GDP) of country $i$ and $j$, and $Ni$ and $Nj$ represents the populations in $i$ and $j$. $Di,j$ and $Pi,j$ are the distance variables that are respectively the physical distance and a potential special preference relationship between $i$ and $j$. The bilateral trade gravity model is now the workhorse of applied international economics^21^ and is used in many different contexts. When we assume the population does not have a significant impact (${\beta }_{2}= {\beta }_{4}=0$) the resemblance to Newton’s original Law of Gravity is even clearer^20,22,23^.

Gravity models are useful when predicting bilateral flows, but face difficulties when it comes to estimating throughflows. Throughflows are extensively estimated in physics and geology to answer questions like how much water is flowing through a specific soil, or through the ocean. Measurements like volumetric or mass flow rates, such as liters per second or kilograms per second, are complemented by optical flow meters that use light to determine flow rates^24^. In social sciences, throughflows are mostly studied as ‘transit’ in the transport literature, much less so in other related fields like migration and trade. It is inflows and outflows, exports and imports, which are topics in the trade and tourism literature, but not so much throughflows see e.g.^21^.

This paper uses a unique dataset on transactions suspicious of money laundering. This proxy for laundering on the left-hand side of the gravity equation allows us to estimate the relevant coefficients on the right-hand side (the country preferences of money launderers). In the Walker/Unger models, the left-hand side was calculated by assuming the weights/coefficients on the right-hand side of the model^7,8^. Our empirical estimations on the decisions of money launderers when choosing the destination of their money allow us to simulate money laundering iteratively flows around the world, hence beyond the first step inspired by^25^. Our estimations, therefore, differentiate between domestic money laundering, money only flowing through and international money laundering settling down in a country.

This paper is structured as follows: “Conceptual framework” section explains our conceptual framework. “Data” section describes our dataset and “Econometric results for money laundering gravity model” section reveals the econometric results of our gravity model. In “Modeling international flows” section we develop a model to use these insights to simulate money laundering flows around the world. “Simulation results” section shows our simulation results. The last section concludes.

## Conceptual framework

When a criminal is so successful that he makes more money than he regularly spends on consumption, he is faced with the issue of money laundering. The criminal will have to make sure that the link between himself, the money and the crime becomes blurred so that he can freely spend his criminal proceeds without being detected by the authorities later.

Legal definitions generally use a very broad definition of money laundering, which even consider situations in which no actual money laundering activities have taken place also as money laundering, such as when the criminal merely has the criminal assets in his possession. This is, for example, the case in the Netherlands^26^, the country from which we got the data for this paper. This would theoretically mean that each economic criminal activity is directly also money laundering. Stealing a candy bar in a shop would then also be money laundering since the criminal possesses the stolen candy bar. However, in practice, one would not consider this money laundering in the narrow and policy-relevant view. Our focus here is to model money laundering activities. We, therefore, apply a narrower definition of money laundering that is more standard in academic writing on money laundering. Money laundering are activities with the goal to hide the criminal origin of the money. The data on which we base our estimates^5^ also uses this narrower definition. Another issue with the definition of money laundering is the relevant predicate offenses that differ per country see^27^. This paper uses an all crimes approach in this respect.

There are many ways to launder money see^28^ for a contemporary overview. The different money laundering methods can roughly be divided into domestic and international money laundering methods. A classic example of domestic money laundering is adding cash to the cash registry of a cash-intensive business (such as a bar, restaurant or, referring to the origin of the name, launderettes^29^). An alternative to laundering money domestically is the standard international money laundering procedure in which the criminal brings the money to a bank and makes it flow internationally through the financial system with (offshore) companies and financial instruments that make it hard to trace the money back to the origin. International money laundering can also be done by smuggling the (cash) money over the border see e.g.^30^ or trade-based money laundering^20^, see e.g.^31^. The advantage of using an international money laundering method is that foreign authorities generally have less knowledge about the criminal and his activities^28^. The disadvantage is the potential detection at the border, or at the bank where the money is first deposited. Hence, the first decision for a money launderer is whether he wants to use a domestic or international money laundering method.

If a money launderer decides to pursue an international money laundering method, he must decide which country or countries to send the money to. When talking about an international money laundering method, we generally consider money laundering through the financial sector (see Supplementary Appendix 1 for some cases that can function as examples of how money is laundered internationally).

Although each money launderer makes his own decision whether to launder domestically or internationally and though it might be hard to understand all decisions in the money laundering process, we can understand the overall pattern from a macro-economic perspective. We start with using a gravity estimation model to get an understanding of how money laundering flows can be explained.

## Data

We accessed and constructed a unique dataset to unveil which (type of) countries money launderers are interested in when deciding where to send their money. We did this with the help of infobox Criminal and Unexplained Wealth (iCOV). iCOV is a Dutch cooperation network of National Police, Tax Office, Customs, Financial Police, Central Judicial Collection Agency, Financial Intelligence Unit, special law enforcement agencies, and the Public Prosecutors Office.

Our dataset contains all STRs (Suspicious Transaction Reports) that were filed in the Netherlands from 2009 to 2014. According to international anti-money laundering standards, obliged entities (such as banks, notaries, accountants and dealers in high-value goods) have to file a report to the Financial Intelligence Unit (FIU) in their country, when they encounter unusual or suspicious transactions in their business. In some countries, obliged entities report Suspicious Transactions (e.g. in Germany). In the Netherlands, obliged entities send so-called Unusual Transaction Reports (UTRs) to their FIU, which then filters out which of these reports are suspicious and need to be passed on to the law enforcement agencies (i.e. which UTRs are STRs). The FATF reasons that reported UTRs on subjective grounds (as to be reported in the Netherlands) are internationally an equal understanding to STRs used by other FIUs based on c.16. 1, 1115. We think that these double-filtered and well-analyzed transactions are the best available proxy for money laundering transactions.

We gained access to all STRs from 2009 to 2018 in the Netherlands, but omitted the last 4 years of STRs from our dataset. This is done because more recent STR data may always have a bias because the investigation of the FIU and the matching with other data takes some time. When a criminal gets arrested for a crime for the first time, he or she gets a criminal record. This new information can imply that some of his/her transactions from the past couple of years will be seen as more suspicious now, too. An arrest in 2019 can lead to transactions (UTRs) from 2016 being re-labeled as suspicious (STRs) in 2016. It, therefore, takes some years for the set of STRs to become a steady and reliable set of data. We analyzed the development of the data and concluded that with a lag of 3 years the data becomes sufficiently reliable. This assumption was confirmed by the iCOV team.

We had the opportunity to use either the STR database or the UTR database. The trade-off here is between having data closer to the criminal behavior we want to analyze (UTR data) and data further from the source but better-quality data (STR data). We prefer to use the STR database instead of the UTR database. The data in the STR database is of higher quality because the Dutch FIU went over these transactions and in the process cleaned up input inconsistencies of reporting entities. Due to these input inconsistencies, the amount of money involved in UTRs is not always known and could therefore not be reliably aggregated. We do however have the number of UTRs from and to the Netherlands and use these as a robustness test. A robustness test (see Table A2 in Supplementary Appendix 3) shows that the results are generally not significantly different.

Our model focuses on international transactions, so we focus on the origin and destination of each transaction. We aggregated, per year for each country pair, the number of STRs, and how much money was involved. We, therefore, ended up with a database of how many STRs and how many suspicious money flows from each country to the Netherlands (and vice versa). This data allows us to identify the importance of the characteristics of the origin countries (the push factors), the characteristics of the destination countries (the pull factors), and the bilateral characteristics (the distance/preference relation factors).

In the next section we try to answer the question: Why would a money launderer send his/her money to one country and not to another one? This is a fundamental question underlying the estimations of the Walker and Unger model and, more generally, all gravity models. Certain countries can appear more attractive to a money launderer than other countries. Country characteristics (such as the criminal justice system, the extent of enforcement) and the distance (between the country of origin and destination) influence the costs and benefits of money laundering. The general hypothesis in gravity models is that flows are larger between larger and closer countries. We complement this hypothesis of the determinants of criminal money flows with the idea that hiding money is easier in a bigger pool of money and that countries closer by (physically as well as culturally) make money laundering easier/cheaper see also^20^. Furthermore, we add specific country characteristics relevant for laundering (such as the degree of corruption and Egmont membership, a multilateral cooperation agreement among Financial Intelligence Units to jointly fight money laundering) to improve the explanations of money laundering flows.

We select relevant determinants (explanatory variables) of money laundering flows based on previous gravity models for money laundering^7,8,12,20^. These can be categorized as country characteristics or distance and preference relationships. GDP serves as the economic mass in our gravity model (World Bank 2019). Wealthier countries may be more attractive for money laundering because they have better developed financial services. We include GDP per capita to capture this effect. We include Egmont membership to control for the effects of better cooperation between national FIUs. Countries that are involved in armed conflicts may be unattractive for money laundering because there is an increased risk of losses. Therefore, we add a variable that measures the magnitude of “Major Episodes of Political Violence” (Center for Systemic Peace 2017). Countries with high corruption can be either more or less attractive for money laundering. On the one hand, bribes add additional costs to the money laundering process. On the other hand, it may be easier to avoid detection in corrupt countries see also^32^. We, therefore, include control of corruption from the Worldwide Governance Indicators in the model. Tax havens may be more attractive for money laundering because they cause lower tax costs. However, tax havens are not well defined. We, therefore, review thirteen academic papers that classify countries as tax havens and count how often each country is designated as tax haven^33–45^. We thank Petr Jansky and Miroslav Palansky for providing the aggregated data.

The distance variables measure the physical and cultural distance between any two countries. From the CEPII dataset, we retrieve the physical distance in kilometers between the population-weighted centers for the countries and dummies for countries that use a common currency, have a colonial background, common religion and common language. To control for the distance between neighboring countries we use a border dummy. Finally, we add the annual value of bilateral trade (Correlates of War 2016). Table 1 presents the summary statistics of the dataset.

**Table 1:** Summary statistics.

Variable	N. obs	Mean	SD	Min	Q25	Median	Q75	Max
**Dependent variables**								
Value of STRs	2952	593,763.883	10,522,037.140					
Number of STRs	2952	26.297	118.025					
Number of UTRs	2952	97.170	610.631					
**Distance variables**								
Border	366,048	0.010	0.099	0.000	0.000	0.000	0.000	1.000
Common language	366,048	0.142	0.349	0.000	0.000	0.000	0.000	1.000
Common currency	366,048	0.017	0.130	0.000	0.000	0.000	0.000	1.000
Colonial history	366,048	0.008	0.090	0.000	0.000	0.000	0.000	1.000
Common religion	366,048	0.133	0.211	0.000	0.016	0.051	0.127	1.000
Physical distance (km)	293,040	8490.814	4691.253	0.995	4773.759	8076.263	12,007.251	19,888.656
Trade (billions US$)	366,048	0.542	7.624	0.000	0.000	0.000	0.003	655.808
**Country characteristics**								
GDP (billions US$)	1209	353.718	1387.466	0.027	5.574	25.130	183.310	17,521.747
GDP pc (US$)	1209	15,817.692	22,972.802	209.854	1792.038	5953.794	19,259.587	179,308.076
Egmont	1482	0.512	0.500	0.000	0.000	1.000	1.000	1.000
Conflict	1482	0.343	1.180	0.000	0.000	0.000	0.000	7.000
Corruption control	1255	− 0.008	0.998	− 1.773	− 0.754	− 0.262	0.767	2.446
Tax haven	1482	2.154	3.885	0.000	0.000	0.000	2.000	13.000

The number of observations for the dependent variables, distance variables and country characteristics represent the number of STR/UTR data points, the total number of bilateral relationships and the number of countries, respectively. Data is for the years 2009–2014. Some descriptive statistics for the dependent variables are omitted because this is confidential data.

## Econometric results for money laundering gravity model

We estimate the money laundering gravity model using the variables described above for two dependent variables, the total value of STRs, and the number of STRs. The selection of independent variables is based on earlier applications of the gravity model to money laundering^7,8,12,14^, even though these applications never actually estimated the gravity model for money laundering (see Tables A3, A4 in Supplementary Appendix 3 for the correlations of these variables). Since we eventually want to simulate money laundering flows around the world, we select only variables that are available for almost all countries and leave out potentially interesting variables, such as bank secrecy/financial secrecy. Arguably the best data on secrecy would be (parts of) the Financial Secrecy Index of Tax Justice Network. Unfortunately, the 2013 edition, the most recent edition in our data period, is available for only 82 jurisdictions. This would mean we would have to drop more than half of the countries from our analysis. Further research with a focus solely on understanding the behavior of money launderers could include such potential interesting determinants, but for this paper, we restrict ourselves to the variables for which we have data for almost all countries in the world. We take the natural logarithm of all variables to make the model multiplicative in line with^20^:

2 $$\text{ln}\,{STR}_{ij}^{v,n}\,=\,{\beta }_{0}\,+\,{{\beta }_{1}\,\text{ln}\,{Border}_{ij}+{{\beta }_{2}\,\text{ln}\,{Language}_{ij}+\beta }_{3}\,\text{ln}\,{Currency}_{ij}+{\beta }_{4}\,\text{ln}\,{Colonial}_{ij}+{\beta }_{5}\,\text{ln}\,{Religion}_{ij}+{\beta }_{6}\,\text{ln}\,{Distance}_{ij}+{\beta }_{7}\,\text{ln}\,{Trade}_{ij}+\beta }_{8}\,\text{ln}\,gd{p}_{i}+{{\beta }_{9}\,\text{ln}\,gd{p}_{j}+\beta }_{10}\,\text{ln}\,gdpp{c}_{i}+{\beta }_{11}\,\text{ln}\,gdpp{c}_{j}+{\beta }_{12}\,\text{ln}\,egmon{t}_{i}+{\beta }_{13}\,\text{ln}\,egmon{t}_{j}+{\beta }_{14}\,\text{ln}\,conflic{t}_{i}+{\beta }_{15}\,\text{ln\,}conflic{t}_{j}+{\beta }_{16}\,\text{ln}\,corruptio{n}_{i}+{\beta }_{17}\,\text{ln}\,corruptio{n}_{j}+{\beta }_{18}\,\text{ln}\,taxhave{n}_{i}+{\beta }_{19}\,\text{ln}\,taxhave{n}_{j}$$

Table 2 displays the estimation results and the coefficients of the gravity model, using the value of suspicious transactions (Column 1) and the number of suspicious transactions (Column 2) on the left-hand side of the gravity model as a proxy for money laundering. The results are generally in line with our expectations. For example, GDP, which serves as the economic mass in our models, has a positive and significant coefficient for both the origin and destination country in both models. This means that countries with higher GDP both send and receive more criminal money to and from the Netherlands. Moreover, physical distance has the expected negative sign, indicating that physical distance deters flows of criminal money. This result is in line with the trade literature, where distance is seen as a transaction cost^46^. Our robustness analysis showed that using other standard distance measures (such as the physical distance in kilometers between the countries based on the most important cities/agglomerations and the distance between capitals) give very similar results.

**Table 2:** Regression results for estimating Eq. (2).

	Dependent variable	
	Value of STRs	Number of STRs
	(1)	(2)
Border	− 5.677 (1.250)***	− 0.983 (0.631)
Common language	6.428 (1.330)***	3.869 (0.733)***
Common currency	0.143 (0.506)	− 0.055 (0.167)
Colonial background	2.665 (0.958)***	0.204 (0.340)
Common religion	2.118 (0.681)***	1.282 (0.224)***
Distance	− 1.490 (0.124)***	− 0.406 (0.042)***
Trade	0.082 (0.021)***	0.023 (0.005)***
GDP:i	0.749 (0.068)***	0.253 (0.018)***
GDP:j	1.126 (0.069)***	0.401 (0.023)***
GDPpc:i	0.125 (0.154)	0.031 (0.039)
GDPpc:j	− 1.094 (0.137)***	− 0.421 (0.050)***
Egmont member:i	0.759 (0.444)*	− 0.092 (0.111)
Egmont member:j	1.607 (0.416)***	0.539 (0.163)***
Conflict:i	0.773 (0.328)**	0.246 (0.089)***
Conflict:j	0.225 (0.263)	0.321 (0.123)***
Corruption control:i	0.460 (0.672)	0.308 (0.176)*
Corruption control:j	− 0.437 (0.672)	− 0.369 (0.226)
Taxhaven:i	0.217 (0.159)	0.095 (0.042)**
Taxhaven:j	0.258 (0.159)	0.039 (0.053)
(Intercept)	13.824 (1.975)***	4.169 (0.615)***
Year dummies included	Yes	Yes
R^2^	0.482	0.501
Adj. R^2^	0.476	0.496
Num. obs	2266	2266
F statistic	144.109	91.285
RMSE	3.852	1.250

All variables are in logs.

Heteroskedasticity consistent standard errors in parentheses.

***p < 0.01, **p < 0.05, *p < 0.1.

**Table 3:** Money laundering estimates for OECD countries for 2014 after five rounds of international flows.

Country	Laundering of domestic criminal money (A)		Throughflows of criminal money (B)		Laundering of foreign criminal money (C)		Total money laundering (A + B + C)	
	Billion US$	% of GDP	Billion US$	% of GDP	Billion US$	% of GDP	Billion US$	% of GDP
Australia	25.0	1.7	7.0	0.5	1.8	0.1	33.9	2.3
Austria	2.5	0.6	7.7	1.7	0.7	0.2	11.0	2.5
Belgium	8.0	1.5	22.1	4.2	2.5	0.5	32.6	6.1
Canada	19.8	1.1	14.6	0.8	3.5	0.2	37.8	2.1
Chile	0.8	0.3	3.0	1.2	0.9	0.3	4.7	1.8
Czechia	1.1	0.5	1.8	0.9	0.3	0.1	3.1	1.5
Denmark	4.7	1.3	1.6	0.5	0.2	0.0	6.4	1.8
Estonia	0.2	0.6	0.6	2.3	0.1	0.2	0.8	3.1
Finland	2.8	1.0	2.7	1.0	0.2	0.1	5.7	2.1
France	27.9	1.0	41.3	1.4	11.2	0.4	80.4	2.8
Germany	55.5	1.4	12.6	0.3	1.4	0.0	69.6	1.8
Greece	1.0	0.4	1.4	0.6	0.3	0.1	2.7	1.1
Hungary	0.7	0.5	2.8	2.0	0.3	0.2	3.8	2.7
Iceland	0.2	1.2	0.2	1.0	0.0	0.1	0.4	2.3
Ireland	1.0	0.4	5.9	2.3	1.3	0.5	8.2	3.2
Israel	4.0	1.3	9.0	2.9	2.4	0.8	15.4	5.0
Italy	16.6	0.8	10.6	0.5	1.2	0.1	28.5	1.3
Japan	9.6	0.2	5.0	0.1	0.9	0.0	15.4	0.3
Latvia	0.1	0.5	0.7	2.2	0.1	0.3	0.9	3.0
Lithuania	0.2	0.4	0.9	1.9	0.1	0.3	1.3	2.6
Luxembourg	0.5	0.8	2.8	4.2	0.3	0.5	3.6	5.5
Mexico	6.2	0.5	26.7	2.0	8.4	0.6	41.3	3.1
Netherlands	8.8	1.0	4.7	0.5	0.6	0.1	14.1	1.6
New Zealand	3.0	1.5	2.9	1.4	0.7	0.3	6.5	3.2
Norway	11.0	2.2	1.0	0.2	0.1	0.0	12.2	2.4
Poland	4.1	0.8	4.7	0.9	0.6	0.1	9.4	1.7
Portugal	0.9	0.4	7.4	3.2	1.5	0.6	9.7	4.2
Slovakia	0.3	0.3	1.6	1.6	0.2	0.2	2.1	2.1
Slovenia	0.3	0.5	0.8	1.6	0.1	0.2	1.2	2.3
South Korea	1.8	0.1	4.7	0.3	0.7	0.0	7.2	0.5
Spain	6.5	0.5	16.5	1.2	4.7	0.3	27.6	2.0
Sweden	12.7	2.2	2.8	0.5	0.2	0.0	15.8	2.8
Switzerland	11.3	1.6	13.3	1.9	1.6	0.2	26.1	3.7
Turkey	1.1	0.1	10.3	1.1	2.3	0.2	13.7	1.5
United Kingdom	37.2	1.2	74.4	2.5	17.8	0.6	129.4	4.3
United States	157.8	0.9	66.4	0.4	17.9	0.1	242.1	1.4
OECD total	445.1	0.9	392.5	0.8	87.2	0.2	924.9	1.9
World total	540.6	0.7	1433.6	1.8	358.4	0.5	2332.6	3.0

*Source* Estimated by authors. Note that total throughflows (1433.6 billion) are by definition 4 times the total amount of laundering of foreign criminal money (358.4 billion).

## Modeling international flows

To ultimately simulate money laundering across the world, we develop a model for the international flows of criminal money between all countries, as well as the throughflows through all countries. For $n$ countries in our model, indexed $i = 1,2, \ldots ,n$ (when it is the country where the money comes from) and $j = 1,2, \ldots ,n$ (when it is the country where money flows to). We start with the amount of money that is generated by crime in each country $i$ and that needs to be laundered, $c_{i}$. Some of this money is laundered domestically, the rest is laundered internationally. The fraction of domestic laundering will be different for each country. We define $\widehat{{d_{i} }}$ to be the fraction of domestic money laundering for country $i$. By definition, $\widehat{{d_{i} }}  \in \left[ {0,1} \right]\epsilon \forall i$. The amount of money that is laundered in the country where it is generated, $d_{i}$, is the product of $c_{i}$ and $\widehat{{d_{i} }}$:

3 $$ d_{i} = c_{i}  \widehat{{d_{i} }} . $$

By construction, the remaining amount, $c_{i} - d_{i}$, is sent abroad.

An adjacency matrix describes the relative importance of each country for all outflows and determines to which countries money flows from each country. Since all countries are (potentially) both senders and receivers of criminal money, the adjacency matrix is a square $n$ by $n$ matrix:

4 $$ A = \left[ {\begin{array}{*{20}c} 0 & {a_{1,2} } & \cdots & {a_{1,n} } \\ {a_{2,1} } & 0 & {} & \vdots \\ \vdots & {} & \ddots & \vdots \\ {a_{n,1} } & \cdots & \cdots & 0 \\ \end{array} } \right]. $$

In matrix $A$, the rows represent the sending countries and the columns the receiving countries, so that each element $a_{i,j}$ is the percentage of total outflowing money from country $i$ that flows to country $j$. As such, $a_{i,j}$ represents the relative importance of country $j$ for criminal money sent abroad by country $i$. By definition, the sums of the rows must all equal 1 (100%), because all outflowing money from each country needs to go somewhere. The diagonal elements of $A$ are zero because this matrix only describes the relative importance between different countries. The importance of domestic money laundering for a country’s criminals is captured by $\widehat{{d_{i} }}$, defined above.

In each round, there is an amount of money in each country that needs to be sent abroad. In the first round, these are the amounts $c_{i} - d_{i}$. International money laundering generally consists of several steps where the money is sent through multiple countries to hide its criminal origin. We assume that there are a number of rounds where money only flows through countries to disguise its origin but is not yet parked or invested. Therefore, in subsequent rounds, the amount sent abroad by each country equals the amount received in the previous round.

In each round $k = 1,2, \ldots ,K$, the amount of money sent from country $i$ to country $j$ is calculated as

5 $$ f_{i,j}^{\left( k \right)} = \left\{ {\begin{array}{*{20}l} {\left( {c_{i} - d_{i} } \right)a_{i,j} } \hfill & {{\text{if }}k = 1} \hfill \\ {s_{i}^{\left( k \right)} a_{i,j} } \hfill & {{\text{if }}k > 1,} \hfill \\ \end{array} } \right. $$

where

6 $$ s_{i}^{\left( k \right)} \equiv r_{j}^{{\left( {k - 1} \right)}} \epsilon \forall i = j $$

i.e. the amount sent ($s_{i}^{\left( k \right)}$) abroad by country $i$ in round $k$ is equal to the amount received in round $k - 1 \left( {r_{j}^{{\left( {k - 1} \right)}} } \right)$ with

7 $$ r_{j}^{{\left( {k - 1} \right)}} = \mathop \sum \limits_{i = 1}^{n} f_{i,j}^{{\left( {k - 1} \right)}} . $$

Calculating this for all $n\left( {n - 1} \right)$ combinations of countries and $K$ rounds completes the simulation.

We compute the total throughflows of money laundering through country $j$ as the sum of the total inflows of rounds $1$ to $K - 1$:

8 $$ t_{j} = \mathop \sum \limits_{k = 1}^{K - 1} r_{j}^{\left( k \right)} . $$

We assume that money is finally parked (the so called integration phase of money laundering) after $K$ rounds. Therefore, laundering of foreign generated criminal money in country $j$ equals the inflow in round $K$, $r_{j}^{\left( K \right)}$.

We define total money laundering as the sum of laundering domestic criminal money, total throughflows, and the final laundering of criminal money,

9 $$ l_{j} = d_{i} + t_{j} + r_{j}^{\left( K \right)} . $$

**Figure 1:**
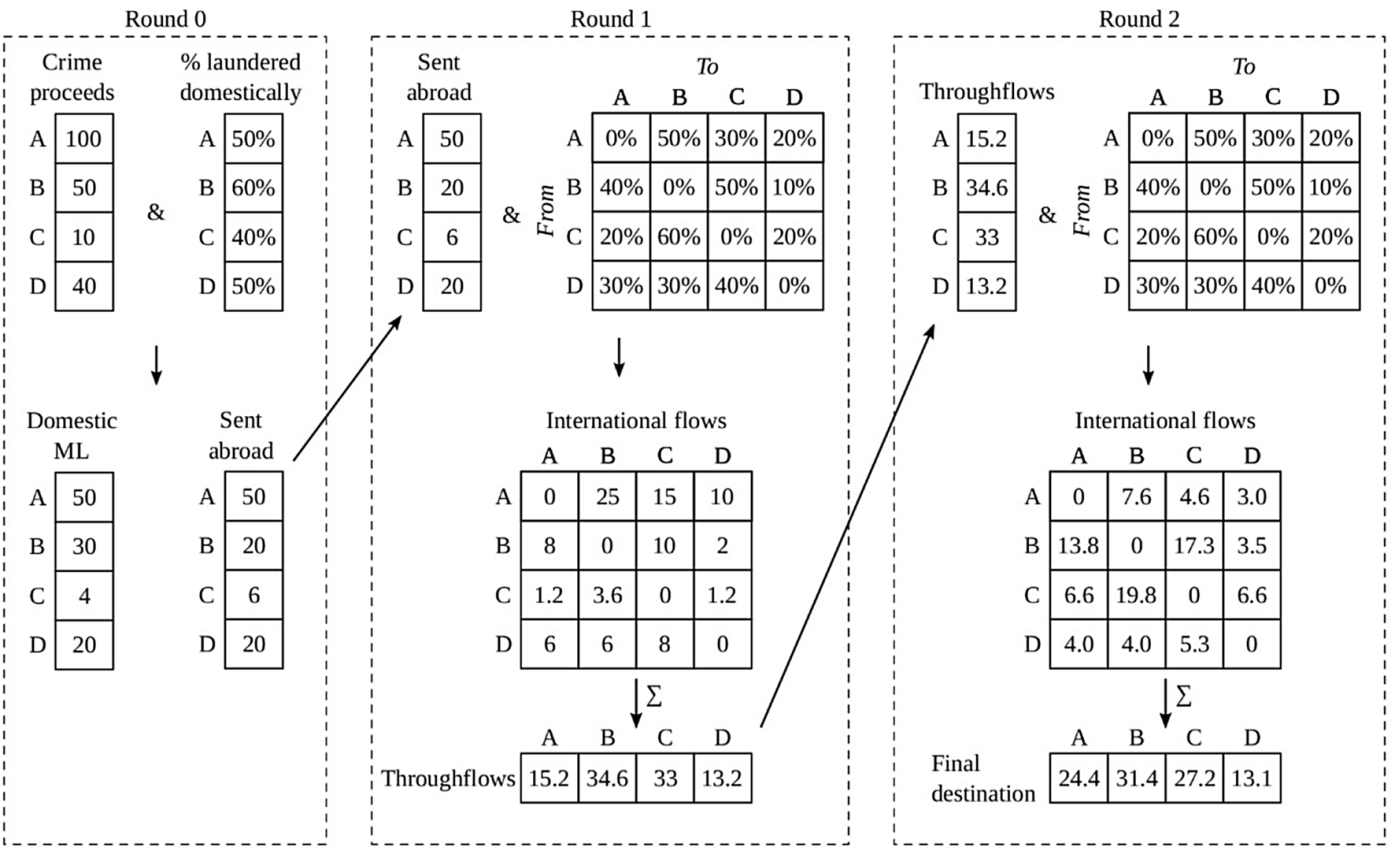
Simplified numerical example of the procedure for four countries and only two rounds of international flows. In round 0, criminals decide whether they launder their criminal money domestically or internationally (related to Eq. (3)). The criminal money that is sent abroad is distributed over the other countries by the international flow percentages matrix (see Eq. (4)). In round 1, the inflows are calculated by taking the sum of the columns of the international flow matrix. This amount is then sent abroad in round 2 (see Eqs. (5)–(7)). In the final round (round 2 in this example) the inflows to each country are laundered in that country. In the actual simulation of this paper, we use five rounds of international flows.

Figure 1 provides a simplified numerical example of the different rounds in our model.

This means that we would be able to simulate how money laundering flows around the world and to which extent if we know the following elements:

- how much criminal money is generated in each country ($c_{i}$),
- what percentage is laundered domestically in each country ($\widehat{{d_{i} }}$), and
- the relative importance of each country for each country ($A$).

Unger et al.^12^ provide estimates for the amount of money generated by crime in each country that needs to be laundered. We convert all amounts to US dollars and use the dollar values for $c_{i}$. The relevant values for $A$ and $\widehat{{d_{i} }}$ are not readily available. We use our dataset presented in “Data” section to produce an estimate for $A$ and $\widehat{{d_{i} }}$.

A limitation of our dataset is that it only has data for transactions to and from the Netherlands. We, therefore, must assume that the same drivers that attract/deter criminal money flowing from and to the Netherlands also hold for attracting/deterring criminal money flowing from and to other countries. We have data for all the country characteristics and all the bilateral physical and cultural distance in our dataset. To find the percentages in matrix $A$, we combine this data with the coefficients found with our gravity model in “Econometric results for money laundering gravity model” section (based on Eq. (2)) and make an out-of-sample estimation of the number and value of all STRs between all countries.

Not all money laundering flows are detected and recorded as an STR, so the STRs in our dataset are only a subset of the actual money laundering flows. This is reflected in the estimated coefficients so that the out-of-sample estimated STRs are a subset of the actual money laundering flows as well. However, we assume that the relative size of the fitted values reflects the actual proportion of money laundering flows. We, therefore, estimate the elements of matrix $A$ as follows:

10 $$ a_{ij} = \frac{{\widehat{STR}_{ij}^{v} }}{{2\mathop \sum \nolimits_{j = 1} \widehat{STR}_{ij}^{v} }} + \frac{{\widehat{STR}_{ij}^{n} }}{{2\mathop \sum \nolimits_{j = 1} \widehat{STR}_{ij}^{n} }} $$

where $$\widehat{STR}_{ij}^{v}$$ and $$\widehat{STR}_{ij}^{n}$$ are the dollar value and number of estimated STRs from country $$i$$ to country $$j$$, respectively (by definition, $$\widehat{STR}_{ij}^{v}$$ and $$\widehat{STR}_{ij}^{n}$$ are 0 when ables to make the model multiplicative in li$$ i = j$$). We use the average of the shares in the value and in the number of estimated STRs, instead of choosing either, to limit the outliers that can come from a single large STR or many small STRs for a country pair in our dataset.

We use a separate calculation to estimate the percentage of generated criminal money that each country launders domestically ($\widehat{{d_{i} }}$) since gravity models do not estimate the proximity of a country to itself. For this calculation, we assume that countries that are attractive for money laundering by foreign criminals are also attractive for money laundering by domestic criminals. Supplementary Appendix 2 specifies our estimation procedure for $\widehat{{d_{i} }}$ in detail.

## Simulation results

Following the procedure explained in “Modeling international flows” section, we simulate the international flows between 187 countries. From the cases we provided as examples in Supplementary Appendix 1, we can see that money is pumped around the world several times, but not endlessly. Most cases use four to six different countries. We assume in our simulation model that money is transferred internationally five times. Future research would be needed to determine whether our assumption is valid. However, we perform a robustness analysis to determine how sensitive our results are to this specific assumption. A robustness analysis (see Table A5 in Appendix 3) shows that our results are robust to different assumptions about how often money is transferred internationally (1 to 10 times). Assuming that money is transferred internationally four or six times gives virtually the same results as our current assumption of five times (correlations of > 0.99).

Since we use Dutch Suspicious Transaction Reports (i.e. transactions flowing from other countries to the Netherlands and from the Netherlands to other countries) and assume similar patterns to hold for other countries, our estimates are more realistic for countries similar to the Dutch economy. Hence, it is more likely that our results hold for rich and developed countries than for poor and underdeveloped countries. Therefore, we focus our analysis on the estimates for the 36 OECD countries.

Table displays the amount of money laundering in our simulation for the 36 OECD countries. We recognize that the policy challenges are very different for different types of money laundering. We therefore report the results for (a) the amount of money laundered domestically (i.e. the money that never leaves the country and is related to domestic crime), (b) the throughflows of criminal money (i.e. money that is made with crime abroad merely passing though the country on its way to its final destination), and c) laundering of foreign criminal money (i.e. money that is made with crime abroad and that found its way to its final destination and is not sent any further).

Our simulations show that money laundering generally amounts to a couple of percentages of GDP (1.9% for OECD countries, while the World average is 3%). Relatively small countries seem to be used mostly as throughflow countries. Latvia, Luxembourg, Portugal, Slovakia, Turkey, and Estonia all have more than three-quarters of their money laundering challenge from throughflow. Norway, Sweden, Germany, Australia, Denmark, and the US are on the other end of the spectrum with the main money laundering challenge being the laundering of domestic crime proceeds.

Most money laundering, according to our simulations, happens in the United States and the United Kingdom, together responsible for 40% of all money laundering in the 36 OECD countries. However, when corrected for country size, as a percentage of GDP, the amount of money laundering is highest in Belgium, Luxembourg, and Israel. Japan and South Korea have relatively the smallest money laundering problem.

## Conclusions and discussion

Different amounts and different types of money laundering flows have very different effects and lead to different enforcement challenges. Countries that mainly deal with criminals laundering their proceeds locally need very different measures than countries that mainly deal with foreign illegal investments or dirty money just flowing through the country. We develop and operationalize a theoretical model that allows us to simulate all international money laundering flows.

For our simulations, we need to know how much money needs to be laundered in each country, how much of that is sent internationally, and where this money is then sent to. To estimate this necessary information, this paper accesses and constructs a unique dataset with the help of infobox Criminal and Unexplained Wealth (iCOV), a Dutch network of authorities and agencies. The dataset contains all STRs (Suspicious Transaction Reports) that were filed in the Netherlands from 2009 to 2014. In line with international anti-money laundering standards, Dutch obliged entities (such as banks, notaries, accountants and dealers in high-value goods) have to file a report to the Financial Intelligence Unit (FIU) when they encounter unusual transactions in their business. The FIU then filters out which of these reports are suspicious and need to be passed on to the law enforcement agencies. We think that these double-filtered and well-analyzed STRs are the best available proxy for money laundering transactions.

This paper combines this data with country and distance characteristics to provide two main contributions. First, this paper tries to get a better understanding of what money launderers are looking for when deciding the next destination of the criminal proceeds. Former money laundering estimation models^7,8,12^ used assumptions on which country characteristics money launderers are looking for when deciding where to send their ill-gotten gains. We empirically test these assumptions with a gravity model. The general hypothesis in gravity models is that flows are larger between larger and closer countries. We complement this hypothesis of the determinants of criminal money flows with the idea that hiding money is easier in a bigger pool of money and that countries closer by (physically as well as culturally) make money laundering easier/cheaper see also^20^. We confirm that there are larger money laundering flows between bigger countries (measured in GDP), ceteris paribus. We also confirm that distance is a cost for money laundering and that, therefore, countries closer to each other have larger bidirectional money laundering flows. We also find that cultural closeness, such as speaking a common language and having a common religion facilitate money laundering flows between countries.

We use our understanding of which country characteristics money launderers are interested in for our second contribution: simulating all money laundering flows around the world. This allows us, for the first time, to provide estimates that distinguish between three different policy challenges: the laundering of domestic crime proceeds, international investment of dirty money, and money just flowing through.

Our simulations show that money laundering generally amounts to about 1.9% for OECD countries, while the World average is 3%. Since we use Dutch Suspicious Transaction Reports (i.e. transactions flowing from other countries to the Netherlands and from the Netherlands to other countries) and assume similar patterns to hold for other countries, our estimates are more realistic for countries similar to the Dutch economy. We, therefore, focus our discussion on the results for OECD countries, the 36 richest countries in the world. We see a number of relatively small countries that are mostly used as throughflow countries, such as Latvia, Luxembourg, and Portugal, while countries like Norway, Sweden, Germany, Australia, Denmark, and the US are on the other end of the spectrum with the main money laundering challenge being the laundering of domestic crime proceeds. Laundering of domestic crime as a share of total money laundering is 90% for Norway, 80% for Sweden and Germany, 74% for Australia, 73% for Denmark and 65% for the US.

Most money laundering, according to our simulations, happens in the United States and the United Kingdom, together responsible for 40% of all money laundering in the 36 OECD countries. However, as a percentage of GDP, the amount of money laundering is highest in Belgium, Luxembourg, and Israel. Japan and South Korea have relatively the smallest money laundering problem.

Our simulations are based on a theoretical model and a number of assumptions. This simulation model is the first of its kind and needs to be developed further in the future with better and more data from also other countries in order to test its assumptions. The results of our simulations must, therefore, be seen as preliminary. It could be that some results are not in line with the presumptions in the money laundering field and with other existing money laundering estimates. But it can be seen as a first step worth exploring further. Eventually, triangulation might be needed to get a better understanding of the different estimates.

This paper provides a framework for money laundering estimations. Once more data becomes available, this data can be incorporated in this model to improve the estimations. At the moment, only the Netherlands made data on STRs available for research. We hope that this paper shows why it would be important to get a better understanding of the money laundering problem and identify the specific policy challenges for different countries. We hope that this convinces more countries to make relevant data available so that the estimates can be improved, and underlying assumptions can be scrutinized.

## Supplementary information

Supplementary Information 1.

Supplementary Information 2.

Supplementary Information 3.

Supplementary Information 4.

Supplementary Information 5.

Supplementary Information 6.

Supplementary Information 7.

Supplementary Information 8.

Supplementary Information 9.

Supplementary Information 10.

Supplementary Information 11.
