# Supplementary material for: Estimating money laundering flows with a gravity model-based simulation
Source: Sci Rep. 2020 Oct 29;10:18552. doi: 10.1038/s41598-020-75653-x (PMC7596494; doi:10.1038/s41598-020-75653-x)
Supplement: Supplementary file 10 — Supplementary Information 10. [file 41598_2020_75653_MOESM10_ESM.docx]

Estimating Money Laundering Flows with a Gravity Model-Based Simulation^[[1]](#footnote-1)^*

Joras Ferwerda*^a^, Alexander van Saase^a^, Brigitte Unger^a^, Michael Getzner^b^

^a^ *Utrecht University School of Economics (U.S.E.), Kriekenpitplein 21-22, 3584 EC Utrecht, The Netherlands*

^b^*Vienna University of Technology – TU Wien, Karlplatz 13, 1040 Vienna, Austria*

**Appendix 1**

To make the money laundering method more concrete, we can look at famous money laundering cases from the Netherlands as an example. In the Netherlands, Willem Endstra, a famous real estate agent and ‘banker of the underworld’ had confided his money laundering techniques to the police shortly before he was assassinated. The ‘Endstra tapes’ are a unique source of information for money laundering techniques related to real estate. In one case, Willem Holleeder, the kidnapper of Dutch beer brewer Freddy Heineken, extorted 3 million Euros from real estate agent Willem Endstra. A schematic illustration of the money laundering transactions used in this case can be seen in Figure A1.

*Figure A1. Example of a money laundering case using the standard international money laundering method*


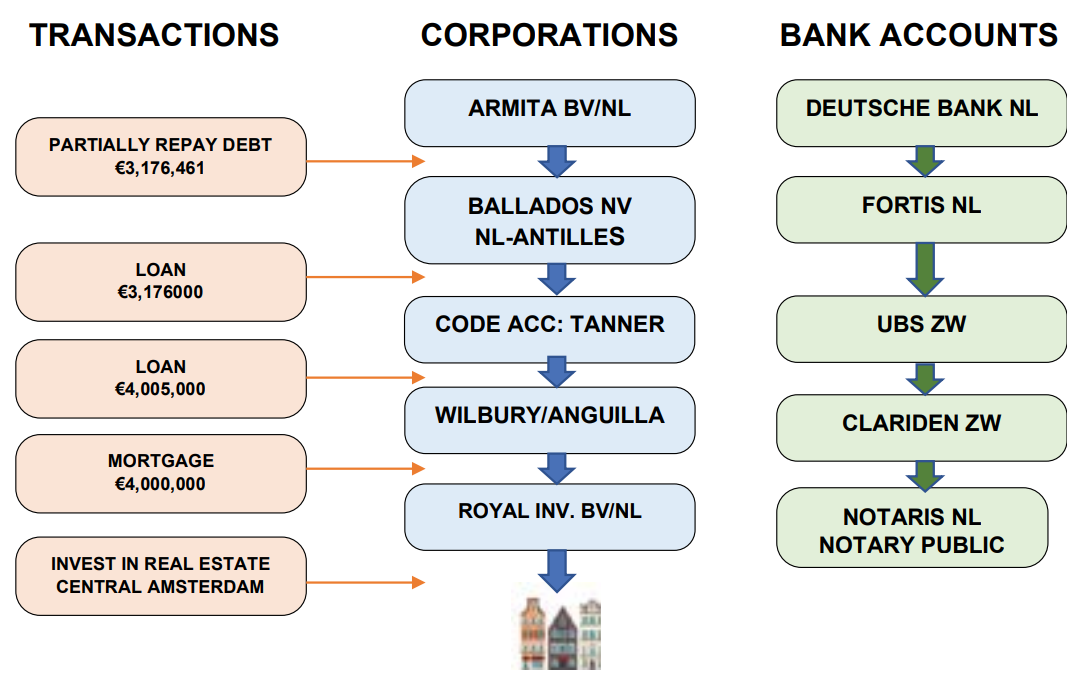


Source: van Koningsveld ^1^, translated graphic ^47^.

In the money laundering case, shown in Figure A1, the money launderer transfers the money through diverse (mortgage) loans to companies from the Netherlands to the Dutch Antilles, then to a code account in Switzerland and then via a company in Anguilla back to the Netherlands to finally park the money in a prestigious real estate object in Amsterdam.

The Financial Action Task Force (FATF) ^48^ lists many more examples of international money laundering methods, we mention here only two cases for illustration purposes. In one case, embezzled public funds in Russia are moved out of sight using companies in Delaware (US), British Virgin Islands, France and Luxembourg and a bank account in Cyprus ^48^. In another case, public officials in Ecuador set up companies in Panama, Hong Kong, British Virgin Islands, Bahamas, Uruguay, and the US to hide bribe payments ^48^. See the full case descriptions below.

“Embezzled public funds worth RUB 300 million (Russian rubles) (USD 11 million) were transferred from the account of Company K to the account of Company R. Company R, a Delaware corporation, was owned and managed by the Russian wife of the suspect, a state official. The same day, Company R transferred USD 11 million as a loan to an account of Company A (BVI) held by a Cypriot bank. Company A then transferred more than USD 11 million to the Company D (US) to purchase real estate in France. Company D transferred more than USD 12 million to a French Notaries Bureau. Information from the FIU of Luxembourg showed that one of the US banks acted as a guarantor for the suspect’s wife in a transaction to purchase of shares of a French company – and the holder of the real estate. The transaction was conducted via an S.S. company – a French subsidiary of a Luxembourg S.D. SA., incorporated and owned by the same individual. Analysis showed that these two chains were interrelated and the real estate was purchased with the proceeds of public funds embezzled for the benefit of the state official’s wife.” ^48^

“Public officials in Ecuador, along with relatives and individuals connected to law firms, created a series of shelf companies in several countries for the purpose of receiving bribe payments. The bribe payments were effected through individuals with links to companies that provide goods and services to a public institution in the oil sector. To send the payments, and to hide the real beneficiaries of the transfers, the suppliers created companies in Panama, Hong Kong, British Virgin Islands, Bahamas, Uruguay, and the US.” ^48^

**Appendix 2**

We use a separate calculation to estimate the percentage of generated criminal money that each country launders domestically ($\overset{̂}{d_{i}}$) since gravity models do not estimate the proximity of a country to itself. For this calculation, we assume that countries that are attractive for money laundering by foreign criminals are also attractive for money laundering by domestic criminals. We estimate a new model that includes only the destination country characteristics:

|  | $\ln{STR}_{ij}^{v}=\beta_{0}+\beta_{1}\ln gdp_{j}+\beta_{2}\ln gdppc_{j}+\beta_{3}\ln egmont_{j}+\beta_{4}\ln conflict_{j}+\beta_{5}\ln corruption_{j}+\beta_{6}\ln taxhaven_{j}$ | (A2.1) |
| --- | --- | --- |

Based on the estimated coefficients and the minimum and maximum value of each variable in the dataset we calculate a theoretical minimum and maximum of the LHS of the equation above. To get the maximum of the LHS of the equation we multiply the positive coefficients with the maximum of the variable and the negative coefficients with the minimum of the variable, and vice versa for the minimum of the LHS.

|  | $\hat{max\{\ln{STR}_{ij}^{v}\}}=\hat{\beta_{0}}+\hat{\beta_{1}}\max\left\{ \ln gdp_{j} \right\}+\hat{\beta_{2}}\min\left\{ gdppc_{j} \right\}+\hat{\beta_{3}}\max\{\ln egmont_{j}\}+\hat{\beta_{4}}\max\left\{ \ln conflict_{j} \right\}+\hat{\beta_{5}}\min\left\{ \ln corruption_{j} \right\}+\hat{\beta_{6}}\min\left\{ \ln taxhaven_{j} \right\}$ | (A2.2) |
| --- | --- | --- |
|  | $\hat{min\{\ln{STR}_{ij}^{v}\}}=\hat{\beta_{0}}+\hat{\beta_{1}}\min\left\{ \ln gdp_{j} \right\}+\hat{\beta_{2}}\max\left\{ gdppc_{j} \right\}+\hat{\beta_{3}}\min\{\ln egmont_{j}\}+\hat{\beta_{4}}\min\left\{ \ln conflict_{j} \right\}+\hat{\beta_{5}}\max\left\{ \ln corruption_{j} \right\}+\hat{\beta_{6}}\max\left\{ \ln taxhaven_{j} \right\}$ | (A2.3) |

The maximum value represents the amount a hypothetical country that is the most attractive for money laundering would receive. The minimum value is what the hypothetical least attractive country would receive.

For each country, we calculate the percentages for domestic money laundering by linearly rescaling its fitted value to a scale of 0 to 100% based on the theoretical minimum and maximum:

|  | $d_{j}=\frac{\hat{\ln{STR}_{ij}^{v}}-\hat{min\{\ln S_{ij}^{v}\}}}{\hat{max\{\ln{STR}_{ij}^{v}\}}-\hat{min\{\ln S_{ij}^{v}\}}}$ | (A2.4) |
| --- | --- | --- |

Table A1 shows the regression results of a model that only includes the destination country characteristics. The results are comparable to those in Table 2, except for the negative coefficient on Tax Haven. The coefficients shown in Table A1 are used in equation A2.2 and A2.3 to estimate for each country the percentage of criminal money generated in each country that stays in that country as specified in equation A2.4.

*Table A1 Regression results for a model including only the destination country characteristics.*

|  | *Dependent variable:* |
| --- | --- |
|  | Value of STRs |
|  | (1) |
| GDP:j | 0.850 (0.059)^***^ |
| GDPpc:j | -0.456 (0.154)^***^ |
| Egmont member:j | 3.946 (0.470)^***^ |
| Conflict:j | 0.328 (0.294) |
| Corruption control:j | -4.980 (0.732)^***^ |
| Tax haven:j | -0.636 (0.186)^***^ |
| (Intercept) | 11.494 (0.897)^***^ |
| R^2^ | 0.146 |
| Adj. R^2^ | 0.144 |
| Num. obs. | 2266 |
| F statistic | 128.971 |
| RMSE | 4.925 |
| Heteroskedasticity consistent standard errors in parentheses.  ^***^p < 0.01, ^**^p < 0.05, ^*^p < 0.1 | |

**Appendix 3**

*Table A2 Comparison of the regression results for the number of STRs and the number of UTRs for 2014. The dependent variables are rescaled to a range of 0 to 1 to allow for comparison of the coefficients between models.*

|  | Number of STRs | Number of UTRs | Z-score |
| --- | --- | --- | --- |
|  | (1) | (2) |  |
| Border | -0.187 (0.218) | -0.210 (0.121)^*^ | 0.094 |
| Common language | 0.621 (0.240)^**^ | 0.658 (0.131)^***^ | -0.135 |
| Common currency | -0.030 (0.054) | -0.032 (0.041) | 0.034 |
| Colonial background | 0.038 (0.120) | 0.055 (0.069) | -0.126 |
| Common religion | 0.124 (0.077) | -0.014 (0.066) | 1.369 |
| Distance | -0.084 (0.014)^***^ | -0.075 (0.011)^***^ | -0.536 |
| Trade | 0.005 (0.002)^***^ | 0.004 (0.002)^*^ | 0.399 |
| GDP:o | 0.035 (0.006)^***^ | 0.056 (0.006)^***^ | -2.297^**^ |
| GDP:d | 0.054 (0.008)^***^ | 0.068 (0.006)^***^ | -1.434 |
| GDPpc:o | 0.011 (0.013) | -0.009 (0.014) | 1.057 |
| GDPpc:d | -0.070 (0.017)^***^ | -0.101 (0.013)^***^ | 1.425 |
| Egmont member:o | -0.035 (0.039) | -0.001 (0.042) | -0.582 |
| Egmont member:d | 0.051 (0.056) | 0.058 (0.042) | -0.111 |
| Conflict:o | 0.058 (0.031)^*^ | 0.015 (0.031) | 0.961 |
| Conflict:d | 0.071 (0.039)^*^ | -0.021 (0.028) | 1.924^*^ |
| Corruption control:o | 0.056 (0.057) | -0.000 (0.059) | 0.684 |
| Corruption control:d | 0.013 (0.080) | -0.026 (0.067) | 0.379 |
| Taxhaven:o | 0.012 (0.013) | 0.011 (0.013) | 0.044 |
| Taxhaven:d | 0.000 (0.018) | 0.014 (0.013) | -0.627 |
| (Intercept) | 0.948 (0.216)^***^ | 1.463 (0.170)^***^ | -1.872^*^ |
| R^2^ | 0.542 | 0.655 |  |
| Adj. R^2^ | 0.518 | 0.637 |  |
| Num. obs. | 378 | 378 |  |
| F statistic | 27.687 | 49.337 |  |
| RMSE | 0.176 | 0.149 |  |
| Heteroskedasticity consistent standard errors in parentheses. ^***^p < 0.01, ^**^p < 0.05, ^*^p < 0.1. | | | |

*Table A3 Correlation matrix for the country characteristics variables, calculated from the average value for each country over the years 2009-2014.*

|  | GDP | GDPpc | Egmont | Conflict | Corruption control | Tax haven |
| --- | --- | --- | --- | --- | --- | --- |
| GDP | 1.000 |  |  |  |  |  |
| GDPpc | 0.188 | 1.000 |  |  |  |  |
| Egmont | 0.150 | 0.368 | 1.000 |  |  |  |
| Conflict | 0.115 | -0.149 | -0.030 | 1.000 |  |  |
| Corruption control | 0.199 | 0.739 | 0.452 | -0.301 | 1.000 |  |
| Tax haven | -0.010 | 0.386 | 0.437 | -0.122 | 0.457 | 1.000 |

*Table A4 Correlation matrix for the dependent variables and distance variables, calculated from the average variable for each country-pair over the years 2009-2014*

|  | Value of STRs | Number of STRs | Number of UTRs | Border | Common language | Physical distance | Colonial history | Common currency | Common religion | Trade |
| --- | --- | --- | --- | --- | --- | --- | --- | --- | --- | --- |
| Value of STRs | 1.000 |  |  |  |  |  |  |  |  |  |
| Number of STRs | 0.889 | 1.000 |  |  |  |  |  |  |  |  |
| Number of UTRs | 0.813 | 0.952 | 1.000 |  |  |  |  |  |  |  |
| Border | -0.001 | 0.042 | 0.119 | 1.000 |  |  |  |  |  |  |
| Common language | -0.004 | 0.098 | 0.178 | 0.088 | 1.000 |  |  |  |  |  |
| Physical distance | -0.078 | -0.124 | -0.152 | -0.175 | -0.011 | 1.000 |  |  |  |  |
| Colonial history | -0.004 | 0.051 | 0.077 | 0.091 | 0.123 | -0.045 | 1.000 |  |  |  |
| Common currency | 0.143 | 0.145 | 0.143 | 0.102 | 0.066 | -0.191 | 0.033 | 1.000 |  |  |
| Common religion | 0.055 | 0.079 | 0.048 | 0.113 | 0.175 | -0.170 | 0.052 | 0.163 | 1.000 |  |
| Trade | -0.003 | 0.083 | 0.144 | 0.154 | 0.008 | -0.058 | 0.041 | 0.060 | 0.012 | 1.000 |

*Table A5 Correlations for the total amount of money laundering per country for different assumptions about how often money is sent around the world (1-10)*

| *Nr of Transfers* | *1* | *2* | *3* | *4* | *5* | *6* | *7* | *8* | *9* | *10* |
| --- | --- | --- | --- | --- | --- | --- | --- | --- | --- | --- |
| 1 | 1.000 |  |  |  |  |  |  |  |  |  |
| 2 | 0.983 | 1.000 |  |  |  |  |  |  |  |  |
| 3 | 0.949 | 0.991 | 1.000 |  |  |  |  |  |  |  |
| 4 | 0.916 | 0.974 | 0.996 | 1.000 |  |  |  |  |  |  |
| 5 | 0.887 | 0.956 | 0.987 | 0.998 | 1.000 |  |  |  |  |  |
| 6 | 0.863 | 0.940 | 0.978 | 0.993 | 0.999 | 1.000 |  |  |  |  |
| 7 | 0.843 | 0.927 | 0.969 | 0.988 | 0.996 | 0.999 | 1.000 |  |  |  |
| 8 | 0.826 | 0.915 | 0.961 | 0.983 | 0.993 | 0.998 | 1.000 | 1.000 |  |  |
| 9 | 0.812 | 0.904 | 0.954 | 0.978 | 0.990 | 0.996 | 0.998 | 1.000 | 1.000 |  |
| 10 | 0.800 | 0.895 | 0.948 | 0.973 | 0.986 | 0.993 | 0.997 | 0.999 | 1.000 | 1.000 |

1. * This paper has been prepared in the Spatial and Network Analysis of Tax Evasion (SPAN) project, financed by infobox Crimineel en Onverklaard Vermogen (iCOV), a Dutch cooperation network of National Police, Tax Office, Customs, Financial Police, CJIB, Financial Intelligence Unit, special law enforcement agencies, the Public Prosecutors Office, Authority for Consumers and Markets and the Dutch Central Bank. Corresponding author is Joras Ferwerda ([j.ferwerda@uu.nl](mailto:j.ferwerda@uu.nl)). [↑](#footnote-ref-1)
